# Estimation of Functional Aerobic Capacity Using the Sit-to-Stand Test in Older Adults with Heart Failure with Preserved Ejection Fraction

**DOI:** 10.3390/jcm11102692

**Published:** 2022-05-10

**Authors:** Iván José Fuentes-Abolafio, Adrian Escriche-Escuder, María Rosa Bernal-López, Ricardo Gómez-Huelgas, Michele Ricci, Manuel Trinidad-Fernández, Cristina Roldán-Jiménez, José María Arjona-Caballero, Antonio Ignacio Cuesta-Vargas, Luis Miguel Pérez-Belmonte

**Affiliations:** 1 Grupo de Investigación Clinimetría CTS-631, Departamento de Fisioterapia, Universidad de Málaga, 29071 Malaga, Spain; ijfabolafio@gmail.com (I.J.F.-A.); adrianescriche@gmail.com (A.E.-E.); manutf35@gmail.com (M.T.-F.); cristina.roldan005@gmail.com (C.R.-J.); jmarjonac@gmail.com (J.M.A.-C.); 2 Instituto de Investigación Biomédica de Málaga (IBIMA), 29590 Malaga, Spain; robelopajiju@yahoo.es (M.R.B.-L.); ricardogomezhuelgas@hotmail.com (R.G.-H.); michele.ricci4@gmail.com (M.R.); luismiguelpb@hotmail.com (L.M.P.-B.); 3 Departamento de Medicina Interna, Hospital Regional Universitario de Málaga, 29010 Malaga, Spain; 4 CIBER Fisio-Patología de la Obesidad y la Nutrición, Instituto de Salud Carlos III, 28029 Madrid, Spain; 5 Facultad de Ciencias Clínicas, Facultad de Salud, Universidad Tecnológica de Queensland, Brisbane, QLD 4000, Australia; 6 Unidad de Neurofisiología Cognitiva, Centro de Investigaciones Médico Sanitarias (CIMES), Universidad de Málaga (UMA), Campus de Excelencia Internacional (CEI) Andalucía Tech, 29010 Malaga, Spain; 7 Centro de Investigación Biomédica en Red Enfermedades Cardiovasculares (CIBERCV), Instituto de Salud Carlos III, 28029 Madrid, Spain

**Keywords:** functional aerobic capacity, heart failure, older adults, sit-to-stand test, 6-min walking test

## Abstract

**Background**: The 6-Min Walking Test (6MWT) has been proposed to assess functional aerobic capacity in patients with heart failure, but many older adults with heart failure cannot complete it. The adequacy of the 5-repetition Sit-To-Stand (5-STS), a simpler test than 6MWT, to assess the functional aerobic capacity in older adults with heart failure has not been evaluated. **Objectives**: This study aimed to assess the usefulness of 5-STS in estimating maximal oxygen uptake (VO_2_ peak) in older adults with heart failure with preserved ejection fraction (HFpEF). **Methods**: A cross-sectional study was carried out. Patients 70 years and older with HFpEF were included. A bivariant Pearson correlation and subsequent multivariate linear regression analysis were used to analyze the correlations between the 5-STS and the estimated VO_2_ peak. **Results**: Seventy-six patients (80.74 (5.89) years) were recruited. The 5-STS showed a moderate and inversely correlation with the estimated VO_2_ peak (r = −0.555, *p* < 0.001). The 5-STS explained 40.4% of the variance in the estimated VO_2_ peak, adjusted by age, sex, and BMI. When older adults were stratified by BMI, the 5-STS explained 70% and 31.4% of the variance in the estimated VO_2_ peak in older adults with normal weight and overweight/obesity, respectively. **Conclusions**: The 5-STS may be an easy tool to assess functional aerobic capacity in older adults with HFpEF, especially for those with normal weight.

## 1. Introduction

The number of older adults with heart failure (HF) is increasing because of the ageing of the population [1]. The HF prevalence ranges from 1% to 2% in developed countries [1]. HF is also the leading cause of hospital admissions for patients older than 65 years [1] and, along with the other cardiovascular diseases, is the leading cause of disability-adjusted life-years (DALYs) due to non-communicable diseases and the main cause of death, especially in countries with higher life expectancy [1,2,3]. Thus, approximately half of the patients with HF will have a hospitalization one year after diagnosis, and 20% will be readmitted again within the same year [1]. In addition, the HF 5-year mortality rate ranged from 56% to 78% [1]. HF is a chronic and clinical syndrome with symptoms or signs caused by a structural or functional cardiac abnormality and corroborated by elevated natriuretic peptide levels or objective evidence of pulmonary or systemic congestion [4,5]. Approximately half of the patients with HF present a preserved ejection fraction of the left ventricle (HF with preserved ejection fraction (HFpEF)) [1,6]. According to clinical and scientific findings, the primary symptoms of these older adults are also fatigue, dyspnea, and exercise intolerance [7,8].

Exercise intolerance is a key determinant of quality of life and mortality in patients with HF [9,10]. Cardiac function was not correlated to exercise intolerance in patients with HFpEF, while skeletal muscle impairments have been closely associated [7,8,11,12,13]. Previous literature has found accentuated muscle dysfunction, with reduced mitochondrial size in skeletal muscle and increased levels of atrophy genes and proteins, in clinically stable outpatients with HFpEF compared with older adults with HF with reduced ejection fraction (HFrEF) and healthy controls [14]. Previous studies have related this skeletal muscle dysfunction with reduced exercise capacity and quality of life in patients with HFpEF [15,16]. These findings are consistent with the greater difficulty in managing older adults with HFpEF, reluctant to most of the medical treatments [17,18,19]. Thus, it might seem logical that patients with HFpEF show worse physical functional performance and exercise intolerance than patients with HFrEF.

The maximal functional aerobic capacity has been inversely correlated to the severity of HF and has been directly correlated to the prognosis and life expectancy [20]. Maximal oxygen uptake (VO_2_ max) is the gold standard for measuring aerobic exercise capacity and exercise tolerance [21]. In the clinical population, the peak oxygen consumption (VO₂ peak), obtained from a cardiopulmonary exercise test, is often a standard measure of functional aerobic capacity and exercise tolerance [22]. These maximal effort tests must be performed in a controlled setting and under the supervision of trained medical personnel to reduce potential risks [22]. This fact makes their implementation difficult in some clinical settings, for which other alternative functional tests have been proposed to assess cardiovascular functional capacity [23]. Thus, the 6-Min Walking Test (6MWT) has been proposed as a simple, inexpensive, safe, reproducible, and alternative cardiopulmonary exercise test to assess functional aerobic capacity in patients with HF [23,24,25,26,27]. The 6MWT could also predict the prognosis of older adults with HF on the basis of the walked distance [24,25,26,27,28]. The 6MWT distance seems to correlate with the VO_2_ max in older adults with chronic HF who do not walk more than 490 m [23,29]. Moreover, some studies have developed equations to predict VO_2_ from the distance walked during the 6MWT in older adults with HF [22,30,31,32]. However, although 6MWT is often described as better tolerated than other walking tests [33], previous studies showed that many older adults could not complete the test [34]. Likewise, clinical experience shows that a significant part of the older adults with HF, especially those with a more limited exercise capacity such as HFpEF, have difficulty completing the test due to fatigue and other symptoms such as dyspnea.

The sit-to-stand (STS) test is frequently used to assess lower limb muscle strength, power, and even physical functional performance [35,36,37,38]. However, some studies suggest the STS as an alternative test to assess functional aerobic capacity [39,40,41]. In this context, Reychler et al. [41] have shown that the STS test could be an alternative to 6MWT to measure the functional aerobic capacity in patients with chronic obstructive pulmonary disease (COPD). A similar option could also be interesting for patients with HFpEF, using modified versions such as the 5-repetition STS (5-STS) test, a well-studied brief and affordable test for most patients [38,42,43]. This 5-STS has shown to be accurate in predicting a poor 6MWT and exercise intolerance in patients with COPD, similar to the 30 s STS in terms of sensitivity and specificity but showing a better patient experience [44,45]. However, to our knowledge, the relationship between the 5-STS test and the 6MWT in patients with HFpEF has not been reported. Thus, the present study aims to find an easier to complete for patients with HFpEF tool, such as the 5-STS, which could estimate the functional aerobic capacity in these patients.

## 2. Methods and Methods

### 2.1. Design and Participants

A cross-sectional study was conducted between April 2019 and March 2020, including 76 older adults with HFpEF recruited as volunteers from the Heart Failure Unit of the Internal Medicine Department at the Regional University Hospital of Malaga (Spain). Inclusion criteria: (1) patients with HFpEF older than 70 years diagnosed according to the consensus statement of the European Society of Cardiology [5]. Exclusion criteria: (1) patients with a New York Heart Association (NYHA) class = 4; (2) patients hospitalized in the last three months; (3) patients with a score on the Mini-Mental State Examination (MMSE) < 24; (4) patients who were not able to stand up from the chair at least five times or who were not able to walk.

### 2.2. Primary Outcomes 

Patients with HFpEF performed the 5-STS test and the 6MWT. In the 5-STS test, patients should stand up and sit down five times as quickly as possible without using their hands to push up from the chair. Patients were recommended to fold their arms across their chest to avoid using their hands. They started sitting in the middle of a standard chair (43 cm in height), with their back straight and their feet placed on the floor. The back of the chair was stabilized against a wall to ensure safety and stability. The time taken to perform the five repetitions was measured using a stopwatch [37,44,45]. The 5-STS has shown high reliability (intraclass correlation coefficient = 0.87) and a minimal detectable change of 3.12 s in patients enrolled in cardiac rehabilitation [43]. The 6MWT was carried out in a closed corridor of 30 m. Two marks were placed on the ground at a distance of 30 m, and patients walked from one end to the other for 6 min. Patients were instructed to walk as quickly as possible, and they were informed of the time elapsed on each lap. The distance that patients walked for six minutes was recorded [32,46,47]. Heart rate (HR) and oxygen saturation were analyzed at each test’s beginning and end using a pulse-oximeter. Dyspnea and leg fatigue were also assessed using the modified Borg scale at the beginning and the end of the 6MWT [48]. The VO_2_ peak was calculated indirectly from the meters the patients walked during the 6MWT, applying the formula developed by Cahalin et al. [21,31]: VO_2_ peak Max = 0.03 × distance (m) in the 6MWT + 3.98. There is a significant correlation (r = 0.64, *p* < 0.001) between the distance ambulated and the estimated VO_2_ peak [31].

### 2.3. Secondary Outcomes

To characterize the included patients with HFpEF with collected clinical-epidemiological data, we collected the age, gender, educational level, marital status, number of comorbidities, medications, left ventricular ejection fraction (LVEF), NYHA class, number of falls in the last year, and smoking status. We also collected blood and urinary biomarkers and anthropometric data such as weight, height, and body mass index (BMI).

### 2.4. Statistical Method

The sample size was calculated with the G Power 3.1.9.2 software (University of Düsseldorf, Düsseldorf, Germany) following the alternative hypothesis: to detect a moderate correlation (r = 0.6) between 5-STS and the estimated VO_2_ peak [41], considering a significance level of 0.05 (error α < 5%), and statistical power of 0.8 (80%), for which a sample consisting of 76 older adults with HFpEF would be needed. L.M.P.-B. carried out the recruitment in his consultations and made it possible to obtain the estimated sample size.

Qualitative measures were described by an absolute frequency and a percentage. Quantitative measures were reported using the mean, the standard deviation (SD), the maximum, and the minimum. Distribution and normality were determined by one-sample Kolmogorov–Smirnov tests (significance < 0.05). The Pearson correlation coefficient (r) assessed the possible bivariate correlations between the 5-STS and the estimated VO_2_ peak. Bivariate correlation was classified into three categories: poor (r ≤ 0.49), moderate (0.50 ≤ r ≤ 0.74), and strong (r ≥ 0.75). The relationship between the 5-STS and the estimated VO_2_ peak was evaluated using multivariate linear regression analysis. The contribution of the exposures to the model’s predictability was assessed by the coefficient of determination (R^2^). A *p*-value of *p* < 0.05 was considered to be statistically significant. All statistical analyses were conducted using the Statistical Package for the Social Sciences (SPSS) 22.0 for Windows (IBM, Madrid, Spain). Additionally, the resulting models were used to develop a software application to help clinicians easily obtain estimated VO_2_ peak values on the basis of raw patient data (http://ejercicioterapeuticouma.es/funnel-equation/).

### 2.5. Ethical Issues

The study was registered on the ClinicalTrial.gov database as NCT03909919. Ethical approval was obtained from the Provincial Ethics Committee of Malaga, Spain (26032020). The study was carried out in accordance with the Declaration of Helsinki [46] and was implemented and reported according to the Strengthening the Reporting of Observational Studies in Epidemiology (STROBE) Statement [47,48]. All participants in this study signed an informed consent form prior to enrolment.

## 3. Results

### 3.1. Patients’ Characteristics

Seventy-six older adults with HFpEF were voluntarily included in the study, without loss of data from any patient. Participants’ anthropometric, clinical-epidemiological variables and blood and urinary biomarkers are shown in Appendix A. The mean age of included older adults with HFpEF was 80.74 years old, and the mean LVEF was 60.62. A total of 44 of the included older adults with HFpEF (57.89%) were women, and 52 subjects showed a NYHA = 2 (68.42%). Most of the included older adults with HFpEF were overweight (42.10%), and 41 older adults (53.95%) had fallen in the past 12 months. Moreover, the included older adults with HFpEF showed an average of 8.43 comorbidities and took an average of 10.21 drugs every day. The most frequent comorbidities were arterial hypertension (97.40%), dyslipidemia (86.80%), heart valve disease (65.80%), and chronic renal insufficiency (64.59%). Moreover, 71 older adults with HFpEF (93.40%) showed a left ventricular dilatation, while 40 older adults with HFpEF (52.60%) showed left ventricular hypertrophy, and 37 of these older adults (48.70%) reported a left atrial dilatation. The drugs that were taken the most were loop diuretics (86.53%), beta-blockers (72.36%), and angiotensin II receptor blocker (61.84%). Descriptive statistics of the functional tests are reported in Table 1. The mean time that older adults with HFpEF took to perform the 5-STS was 15.92 s (5.80), while these older adults walked an average of 244.67 m (97.03) in the 6MWT, and the estimated VO_2_ peak was 11.32 mL/kg/min (2.91). All patients with HFpEF could complete the 5-STS, but only 57.90% of patients with HFpEF could finish the 6MWT.

### 3.2. Correlations and Multiple Regression

The bivariate correlation between the 5-STS and the estimated VO_2_ peak is shown in Table 2. In summary, the 5-STS showed a significant but moderate and inversely proportional correlation with the VO_2_ peak (r = −0.555, *p* < 0.001).

The summary of the multivariate linear regression models is shown in Table 3. Multivariate linear regression models are shown in Table 4. The 5-STS explained 40.4% of the variance in the estimated VO_2_ peak, adjusted by age, sex, and BMI. However, on the basis of patients’ BMI classification, the 5-STS explained 70% and 34.1% of the variance of the estimated VO_2_ peak in subjects with normal weight and overweight/obesity, respectively.

According to these models, a software application was developed to help clinicians easily obtain estimated VO_2_ peak values on the basis of raw patient data (http://ejercicioterapeuticouma.es/funnel-equation/).

## 4. Discussion

This work aimed to study the level of contribution of 5-STS in estimating functional aerobic capacity in older adults with HFpEF. The main finding was a significant and moderate (r = −0.55) inverse correlation between the estimated VO_2_ peak and the time (in seconds) inverted in the 5-STS in older adults with HFpEF. Additionally, 5-STS explained 40.4% of the variance in the estimated VO_2_ peak, adjusted by age, sex, and BMI.

The mean time patients with HFpEF took to perform the 5-STS was 15.92 s (5.80). However, there were large differences between the minimum (8.46) and maximum (34.01) values. These mean values are slightly higher than the estimated reference values for older adults (12.6 s for people between 70–79 years and 14.8 s for people between 80 and 89 years old) [42]. Compared with a previous study that included older adults with HFpEF, HFrEF, and mid-range ejection fraction (9.88 (8.28–12.55)), the mean values of our study were also higher [49]. Even the 5-STS values shown in the present study were higher than the new values reported in adults older than 80 years (13.25 s) [36]. This elevated time to perform the 5-STS could also have been influenced by the age of included patients burdened due to osteomuscular limits or skeletal muscle dysfunctions [10,11]. Included patients also walked an average of 244.67 m (97.03) in the 6MWT. Age and BMI were associated with the 6MWT [50]. The mean age of included older adults with HFpEF was 80.74 years old, and most of them were overweight (42.10%) or obese (36.84%). Thus, age and BMI could have affected the 6MWT values of our study. However, the distance that included patients with HFpEF walked (244.67 m) is lower than the distance previously found in a large cohort of a cardiovascular health study (344 m) with ages similar to those of the patients included in our study [34]. Thus, the functional limitations of the patients with HFpEF included in our study are clear [34].

The preservation of the ejection fraction in these older adults could suggest that they have a greater capacity than older adults with HFrEF [7,8,14]. However, research and clinical experience show that older adults with HFpEF are patients with more significant functional impairment and complex handling than older adults with HFrEF because no pharmacological treatment has demonstrated any clear prognostic benefits [17,18,19,51]. Thus, the values obtained in the 5-STS and the 6MWT in the present study could be explained by the molecular, mitochondrial, and metabolic abnormalities in skeletal muscle, as well as the increased levels of atrophy genes and proteins, previously found in this population [14]. Given the difficulty of performing direct VO_2_ max or peak measurements, alternative functional tests such as 6MWT have been previously used to estimate these values [23]. However, these estimates involve a measurement error, which should be as small as possible and preferably towards an underestimation, avoiding risks in populations with limited capacities [22]. In fact, some studies have reported that the use of sub-maximal functional tests to estimate VO_2_ max may underestimate the actual values [22,52,53]. In the case of the 6MWT, the correlation of the meters walked with the estimated values of VO_2_ peak ranged between r = 0.29 and r = 0.81 in 16 studies conducted with patients with HF (r = 0.64, *p* < 0.001 using the formula applied in this study) [26]. However, despite obtaining acceptable margins of error, the 6MWT is sometimes still excessive for those older adults with the most limited capacity, so using short tests such as the 5-STS would facilitate the measurement of these values in most clinical contexts [38,42,43]. Thus, all the patients with HfpEF included in the present study could complete the 5-STS, but only 57.90% could finish the 6MWT.

The 5-STS showed a significant and moderate inverse correlation with the estimated VO_2_ peak (r = −0.55, *p* < 0.001). This value could be in accordance with the findings of previous studies, which did not find a strong correlation between functional tests and the VO_2_ peak values obtained in cardiopulmonary exercise tests in patients with HFpEF [54]. However, these results may still suggest the possibility of performing this test, usually performed in a short time, as a substitute for 6MWT in populations unable to walk for several minutes. A similar idea was tested before in another population, such as patients with COPD [41]. In this case, a modified version of the STS (1-min STS) found a moderate correlation between the number of repetitions in this test and the distance walked in the 6MWT (r = 0.71, *p* < 0.001) [41]. In our assumption, using an even shorter version, calculating the time required to perform five repetitions may also have influenced the obtaining of slightly worse results.

The 5-STS explained part of the variance in the 6MWT and the estimated VO_2_ peak, adjusted by age, sex, and BMI (40.4%). However, these values differed when older adults were fragmented on the basis of their BMI. The 5-STS shared 70% of the variance of estimated VO_2_ peak in older adults with HFpEF with a normal weight. These values were lower for participants with overweight/obesity (31.4%). These findings could suggest that the 5-STS represents an interesting option, especially in older adults with a normal weight. These differences could also be explained by disparities in performance between the different subgroups. Consistent with this assumption, previous studies found that subjects with obesity had a worse performance than their normal-weight peers in functional tests such as the 5-STS [55] or the 6MWT [56,57]. These differences may influence the idea that markedly longer times in the 5-STS obtain a significantly different correlation with the estimated VO_2_ peak. The VO_2_ peak has also been negatively correlated with the BMI in young adults, patients with HF, and patients with hypertrophic cardiomyopathy, so that obese subjects show a reduced VO_2_ peak compared to normal-weight subjects [58,59,60].

The included older adults with HFpEF showed a low estimated VO_2_ peak (11.32 mL/kg/min), which could be affected by the muscle mass loss associated with the age of the included patients (80.74 years old) [52]. Thus, the simple error of the Cahalin equation (standard error Cahalin et al. ±1 mL/kg/min) could be a source of large variations in the included older adults with HFpEF [17,27]. Furthermore, given the functional limitation of patients with HFpEF and their low VO_2_ peak, the calculation of the VO_2_ peak might not be relevant to some clinicians. However, it might be important for clinicians to find tests that reflect the functional status of their patients. The 5-STS also seems to be a good tool for assessing the frailty (standing up and sitting down can be considered an activity of daily living of the older adults) and the risk of falling [61,62]. To our knowledge, there are no studies in the literature that have analyzed the role of the 5-STS test in older adults with HFpEF. The 5-STS is an easy, reproducible, and largely performing test without the need for advanced staff training or expensive equipment. This 5-STS gives the clinician an option to estimate the peak VO_2_ of their patients, assuming an error, and allows the clinician to know if their patients are frail or at risk of falling.

### 4.1. Implications for Clinical Practice

The present study aims to offer the clinician a tool to estimate the peak VO_2_ of older adults with HFpEF. A calculator has been developed where, on the basis of the time that patients take to perform the 5-STS, age, gender, and BMI, they can directly obtain the estimated VO_2_ peak easily in a short period (http://ejercicioterapeuticouma.es/funnel-equation/).

### 4.2. Future Research

Future research should repeat the present study is a large sample size of patients with HFpEF and patients with HFrEF or other older adults with multiple comorbidities. Future studies also should assess other functional tests easily implemented in a clinical practice, which could estimate the VO_2_ peak in a short period. Moreover, future research should determine the relationship between the 30 s STS or the 1 min STS with the estimated VO_2_ peak since other studies have shown a better relationship between these tests and the estimated VO_2_ peak [39,40,41]. However, older adults with severe functional limitations could not complete one minute STS. A cardiopulmonary exercise test should assess the VO_2_ peak in future studies. Future studies could also assess the STS test using smartphone applications or inertial sensors to quantify temporal variables and other interesting functional kinematic variables during the 5-STS [63]. As BMI affected the 5-STS and 6MWT performance, future studies should assess the relationship between BMI and body composition and physical functional performance in older adults with HFpEF [50,64].

### 4.3. Strengths and Limitations of the Study

One of the strengths of this study is the proposal of a short test that can be performed by older adults with significantly compromised functional capacity. In the case of older adults with HF, their capacity can be overestimated or underestimated if their stratification is taken into account only on the basis of parameters such as the ejection fraction. Thus, older adults with HFpEF present greater impairments than patients with HFrEF, which is why it is necessary to have other functional tests or tests that allow correct stratification. According to the results of this study, 5-STS may be an option to consider along with other existing tests in those patients with HFpEF who are not capable of performing tests of several minutes, especially in older adults with a normal weight. Additionally, providing a software application will allow clinicians to obtain immediately estimated VO_2_ peak values on the basis of crude patient data. Moreover, a large sample of older adults with HFpEF was also recruited to reduce any risk of selection or performance bias. The cross-sectional design could have reduced any risk of detection and attrition bias because it reduced the possibility of missing data. The statistically significant and the statistically non-significant results were presented in this study to avoid publication bias. However, one of the main limitations is using an estimate of the VO_2_ peak through a formula applied to the distance walked on the 6MWT, with an assumed error.

## 5. Conclusions

This study shows that the 5-STS could be useful for assessing the functional aerobic capacity in older adults with HFpEF, especially in normal-weight patients. Older adults with HF show impaired functionality, which has been correlated to the severity of HF and their prognosis, so functional assessment is essential in these patients. However, older adults with HFpEF often show limited exercise capacity and difficulty completing long-term tests due to symptoms such as fatigue and dyspnea. For this reason, clinicians should have short-term tests that allow them to estimate the VO_2_ peak of their patients. More studies showing practical clinical alternatives to estimate VO_2_ peak, the frailty status, and the risk of falling in older adults with HFpEF or HFrEF should be performed.

## Figures and Tables

**Table 1:** Descriptive statistics of the functional tests (n = 76).

			HR_0_	OS_0_	HR_1_	OS_1_	Borg_0_	Borg_1_	Test Completion
	Mean (SD)	Min–Max	Median (Min–Max)	Median (Min–Max)	Median (Min–Max)	Median (Min–Max)	Median (Min–Max)	Median (Min–Max)	N (%)
**5-STS (s)**	15.92 (5.80)	8.46–34.01	78 (45–120)	96 (79–99)	86.50 (51–142)	96 (84–99)			76 (100%)
**6MWT (m)**	244.67 (97.03)	45–540	78 (48–105)	96 (86–99)	96.50 (56–145)	95 (80–99)	0 (0–0)	6 (0–10)	44 (57.90%)
**Estimated VO_2_ peak (mL/kg/min)**	11.32 (2.91)	5.33–20.18							

**SD**: standard deviation; **5-STS**: Five-Repetitions Sit-to Stand; **6MWT**: 6-Min Walking Test; **VO_2_ Peak**: peak oxygen consumption: **HR_0_**: heart rate at the beginning; **OS_0_**: oxygen saturation at the beginning; **HR_1_**: heart rate at the end; **OS_1_**: oxygen saturation at the end: **Borg_0_**: fatigue and dyspnea according to the modified Borg scale at the beginning; **Borg_1_**: fatigue and dyspnea according to the modified Borg scale at the end.

**Table 2:** Bivariate correlation (r, *p*) between the 5-STS and the 6MWT (distance and estimated VO_2_ peak).

	Estimated VO_2_ Peak
**5-STS**	−0.555 **

**5-STS:** Five-Repetitions Sit-to Stand; **6MWT**: 6-Min Walking Test; **VO_2_ Peak**: peak oxygen consumption. ** *p* < 0.001.

**Table 3:** Summary of the multivariate linear regression models of 5-STS.

	R	R^2^	Adjusted R^2^	SE	F	*p*
**Estimated VO_2_ peak**	0.635	0.404	0.370	2.31	12.02	<0.001
	**Normal weight**					
**Estimated VO_2_ peak**	0.837	0.700	0.625	2.01	9.33	0.002
	**Overweight/obesity**					
**Estimated V0_2_ peak**	0.561	0.314	0.278	2.28	8.56	<0.001

**5-STS**: Five-Repetitions Sit-to Stand; **SE**: standard error; **VO_2_ Peak**: peak oxygen consumption.

**Table 4:** Multivariate linear regression models.

Dependent Outcome	Predictor Variables	Non-Standardized Coefficients		Typified Coefficients	t	*p*	95% Confidence Interval (95%CI)
		B	SE	Beta			
**Estimated VO_2_** **peak**	**(Constant)**	25.767	4.746		5.429	0.000	(16.304, 35.231)
	**5-STS**	−0.266	0.047	−0.530	−5.695	0.000	(−0.359, −0.173)
	**Sex**	−1.012	0.553	−0.173	−1.830	0.071	(−2.115, 0.091)
	**Age**	−0.080	0.049	−0.161	−1.622	0.109	(−0.178, 0.018)
	**BMI**	−0.108	0.050	−0.218	−2.174	0.033	(−0.207, −0.009)
**Estimated VO_2_** **peak** **Normal weight**	**(Constant)**	26.939	7.118		3.784	0.003	(11.429, 42.448)
	**5-STS**	−1.047	0.232	−0.775	−4.517	0.001	(−1.552, −0.542)
	**Sex**	−0.898	1.117	−0.142	−0.804	0.437	(−3.332, 1.536)
	**Age**	0.004	0.086	0.008	0.047	0.964	(−0.184, 0.192)
**Estimated VO_2_** **peak** **Overweight/obesity**	**(Constant)**	17.395	4.417		3.938	0.000	(8.546, 26.243)
	**5-STS**	−0.228	0.048	−0.529	−4.703	0.000	(−0.325, −0.131)
	**Sex**	−0.965	0.602	−0.178	−1.602	0.115	(−2.171, 0.242)
	**Age**	−0.026	0.052	−0.057	−0.507	0.614	(−0.130, 0.078)

**SE**: standard error; **95%CI**: 95% Confidence Interval; **VO_2_ Peak**: peak oxygen consumption; **5-STS**: Five-Repetitions Sit-to Stand; **BMI**: Body Mass Index.

## Data Availability

The data presented in this study are available on request from the corresponding author.

## Appendix A

**Table A1:** Participants’ anthropometric, clinical-epidemiological variables, and blood and urinary biomarkers (n = 76).

	Mean (SD)	Min–Max
Age (years)	80.74 (5.89)	70.0–96.0
LVEF (%)	60.62 (4.32)	50.0–75.0
Comorbidities (n)	8.43 (1.90)	4.0–14.0
Height (m)	1.61 (0.08)	1.43–1.84
Weight (kg)	76.54 (15.02)	52.0–116.50
BMI (kg/m^2^)	29.59 (5.88)	19.81–51.78
Polypharmacy (n)	10.21 (3.13)	3.0–19.0
**Blood biomarkers**		
Hb (g/dL)	13.90 (11.12)	6.40–108.0
MCV (fL)	95.65 (19.03)	72–247
Leukocyte count (×10^9^/L)	8.16 (4.83)	2.41–45.20
Blood platelets (×10^9^/L)	220.81 (70.83)	8–415
Glucose (mg/dL)	101.97 (28.51)	61–195
Creatinine (mg/dL)	1.35 (0.82)	0.51–6.33
GF (mL/min/1.73 m^2^)	50.96 (20.36)	8–90
Na^+^ (mEq/L)	139.83 (3.40)	124–148
K^+^ (mEq/L)	4.62 (0.48)	3.5–6.2
Cl^−^ (mEq/L)	101.39 (4.14)	89–111
Glycosylated hemoglobin (%)	6.36 (1.13)	4.8–10.4
Ferritin (ng/mL)	124.46 (139.18)	10–738
Transferrin saturation (%)	21.68 (24.92)	1–214
Total cholesterol (mg/dL)	160.30 (41.48)	98–317
LDL (mg/dL)	86.14 (29.46)	36–167
HDL (mg/dL)	46.62 (14.17)	28–90
NT-proBNP (pg/mL)	2228.72 (3486.43)	42–19118
Vitamin D (ng/mL)	23.67 (10.42)	7–50
Vitamin B12 (pg/mL)	495.12 (641.18)	103–4668
ALT (U/L)	22.08 (13.42)	8–94
CA-125 antigen (U/mL)	43.57 (73.41)	0–525
Folic acid (ng/mL)	8.04 (2.60)	3–16
Thyrotropin (µIU/mL)	2.40 (1.80)	0.1–12
Albumin (g/dL)	3.97 (3.85)	2.30–36
Bilirubin (mg/dL)	0.61 (0.31)	0.1–1.8
**Urinary biomarkers**		
Urine creatinine (mg/dL)	76.55 (34.00)	24.00–158.53
**Drugs**		
ACE inhibitors	16 (21.05%)	
ARB	47 (61.84%)	
Beta-blockers	55 (72.36%)	
Ivabradine	4 (5.26%)	
Ca-antagonists	21 (27.63%)	
Loop diuretics	65 (85.53%)	
Mineralocorticoid receptor antagonist	24 (31.58%)	
Thiazide	10 (13.15%)	
Nitrates	21 (27.63%)	
Acarboxyprothrombin	14 (18.42%)	
Factor Xa inhibitors	32 (42.10%)	
Acetylsalicylic acid	25 (32.89%)	
Hypoglycemic agents	37 (48.68%)	
Metformin	18 (23.68%)	
SGT2I	9 (11.84%)	
GLP1	2 (2.63%)	
DPP4	20 (26.31%)	
Insulin	21 (27.63%)	
**Comorbidities**		
Arterial hypertension	74 (97.40%)	
DM	44 (57.90%)	
Dyslipidemias	66 (86.80%)	
Atrial fibrillation	45 (59.20%)	
COPD	22 (28.90%)	
Stroke	11 (14.50%)	
CRI	49 (64.50%)	
OSA	16 (21.10%)	
Cognitive impairment	9 (11.80%)	
Anemia	37 (48.70%)	
PAD	4 (5.30%)	
Depression	25 (32.90%)	
Cancer disease	12 (15.80%)	
LVH	40 (52.60%)	
LVD	71 (93.40%)	
LAD	37 (48.70%)	
Heart valve disease	50 (65.80%)	
Aortic valve disease	25 (32.90%)	
Mitral valve disease	35 (46.10%)	
Tricuspid valve disease	17 (22.40%)	
Pulmonary hypertension	13 (17.10%)	
**BMI**		
Normal weight	16 (21.05%)	
Overweight	32 (42.10%)	
Obesity	28 (36.84%)	
**Gender**		
female	44 (57.89%)	
male	32 (42.10%)	
**NYHA**		
II	52 (68.42%)	
III	24 (31.58%)	
**History of smoking**		
No	49 (64.47%)	
Yes	27 (35.53%)	
**Number of falls in the last year**		
0	35 (46.05%)	
1	20 (26.31%)	
2	9 (11.84%)	
3	7 (9.21%)	
>3	5 (6.57%)	
**Marital status**		
Single	6 (7.89%)	
Married	32 (42.10%)	
Divorced	1 (1.31%)	
Widower	37 (48.68%)	
**Academic degree/studies level**		
Not knowing how to read or write	11 (14.47%)	
Literacy	34 (44.73%)	
Primary studies	14 (18.42%)	
Secondary studies	6 (7.89%)	
Higher education	11 (14.47%)	

**LVEF:** Left Ventricular Ejection Fraction; **BMI**: body mass index; **Hb**: hemoglobin; **MCV**: mean corpuscular volume; **Na**: sodium; **K**: potassium; **Cl**: chlorine; **GF**: glomerular filtering; **LDL**: low-density lipoprotein cholesterol; **HDL**: high-density lipoproteins cholesterol; **NT-proBNP**: pro-brain natriuretic peptide; **ALT**: alanine aminotransferase; **CA-125 Antigen**: carbohydrate antigen; **ACE inhibitors**: angiotensin-converting enzyme inhibitors; **ARB**: angiotensin II receptor blocker; **SGT2I**: sodium-glucose transporter 2 inhibitors; **GLP1**: glucagon-like peptide 1; **DPP4**: dipeptidyl peptidase-4; **DM**: diabetes mellitus; **COPD**: chronic obstructive pulmonary disease; **CRI**: chronic renal insufficiency; **OSA**: obstructive sleep apnea; **PAD**: peripheral arterial disease; **LVH**: left ventricular hypertrophy; **LVD**: left ventricular dilatation; **LAD**: left atrial dilatation; **NYHA**: New York Heart Association class; **SD**: standard deviation.
